# Dialectical behavior therapy adapted for binge eating compared to cognitive behavior therapy in obese adults with binge eating disorder: a controlled study

**DOI:** 10.1186/s40337-020-00299-z

**Published:** 2020-06-10

**Authors:** Mirjam W. Lammers, Maartje S. Vroling, Ross D. Crosby, Tatjana van Strien

**Affiliations:** 1 Amarum, Expertise Centre for Eating Disorders, GGNet Network for Mental Health Care, Den Elterweg 75, 7207 AE Zutphen, The Netherlands; 2 Radboud University, Behavioural Science Institute, Montessorilaan 3, 6525 HR Nijmegen, The Netherlands; 3 Sanford Center for Biobehavioral Research, Fargo, North Dakota USA; 4 University of North Dakota School of Medicine and Health Sciences, Fargo, North Dakota USA

**Keywords:** Binge eating disorder, Cognitive behavior therapy, Dialectical behavior therapy, Group therapy, Emotion regulation

## Abstract

**Background:**

Current guidelines recommend cognitive behavior therapy (CBT) as the treatment of choice for binge eating disorder (BED). Although CBT is quite effective, a substantial number of patients do not reach abstinence from binge eating. To tackle this problem, various theoretical conceptualizations and treatment models have been proposed. Dialectical behavior therapy (DBT), focusing on emotion regulation, is one such model. Preliminary evidence comparing DBT adapted for BED (DBT-BED) to CBT is promising but the available data do not favor one treatment over the other. The aim of this study is to evaluate outcome of DBT-BED, compared to a more intensive eating disorders-focused form of cognitive behavior therapy (CBT+), in individuals with BED who are overweight and engage in emotional eating.

**Methods:**

Seventy-four obese patients with BED who reported above average levels of emotional eating were quasi-randomly allocated to one of two manualized 20-session group treatments: DBT-BED (*n* = 41) or CBT+ (*n* = 33). Intention-to-treat outcome was examined at post-treatment and at 6-month follow-up using general or generalized linear models with multiple imputation.

**Results:**

Overall, greater improvements were observed in CBT+. Differences in number of objective binge eating episodes at end of treatment, and eating disorder psychopathology (EDE-Q Global score) and self-esteem (EDI-3 Low Self-Esteem) at follow-up reached statistical significance with medium effect sizes (Cohen’s *d* between .46 and .59). Of the patients in the DBT group, 69.9% reached clinically significant change at end of the treatment vs 65.0% at follow-up. Although higher, this was not significantly different from the patients in the CBT+ group (52.9% vs 45.8%).

**Conclusions:**

The results of this study show that CBT+ produces better outcomes than the less intensive DBT-BED on several measures. Yet, regardless of the dose-difference, the data suggest that DBT-BED and CBT+ lead to comparable levels of clinically meaningful change in global eating disorder psychopathology. Future recommendations include the need for dose-matched comparisons in a sufficiently powered randomized controlled trial, and the need to determine mediators and moderators of treatment outcome.

**Trial registration:**

Nederlands Trial Register: NL3982 (NTR4154). Date of registration: 2013 August 28, retrospectively registered,

## Plain English summary

Binge eating disorder (BED) is mostly treated with cognitive behavior therapy (CBT). The treatment focusses on reducing efforts to diet. Yet, a substantial number of patients still suffer from binge eating after this treatment. We suggest that patients with BED are better served with a treatment that helps them cope with negative emotions in a healthier way. Dialectical behavior therapy for BED (DBT-BED) is one such treatment. To test this, we compared outcomes of DBT-BED to the intensive CBT program that is common in our treatment center**.** We did so, in individuals with BED who might especially benefit from DBT-BED: those who are overweight and eat in response to emotions. Greater improvements were observed in the CBT group regarding the number of objective binge eating episodes at the end of treatment, and regarding global eating disorder psychopathology and self-esteem 6 months after treatment. Yet, patients in the CBT group received more therapy hours than in the DBT-BED group, which may have advantaged the CBT treatment. Concurrently, in both groups a comparable percentage of patients showed clinically meaningful changes in global eating disorder psychopathology. In conclusion, our results overall support the intensive CBT program over DBT-BED. Yet, given the fact that DBT-BED is less time-consuming (so cheaper) and presents similar percentages of meaningful change in global eating disorder psychopathology, it is worthwhile to further test the effects of DBT-BED in future studies.

## Introduction

Binge eating disorder (BED) is characterized by psychologically distressing, recurrent, brief episodes of uncontrollable overeating [1]. It is associated with psychiatric comorbidity, impaired social functioning and impaired physical well-being [2, 3]. An estimated 70% of BED patients have a body mass index (BMI) between 30 and 40, and about 20% have a BMI of 40 or higher [4]. While aspects of body-image disturbance are part of the diagnostic criteria for anorexia nervosa and bulimia nervosa, these aspects are not included in the criteria for BED [1]. Nevertheless, several aspects (e.g. body dissatisfaction and the overvaluation of body shape and weight) have shown to be relevant to BED [5]. Current guidelines recommend cognitive behavior therapy (CBT) as the treatment of choice for BED [6, 7]. The most widely supported form of CBT for BED is based on the transdiagnostic model of eating disorders, suggesting that distinctive eating disorders are maintained by similar mechanisms [8]. Clinical perfectionism, interpersonal difficulties, low self-esteem and mood intolerance are acknowledged to act as maintaining factors in many patients. However, the core CBT-protocol focusses on behavior (i.e. dietary restraint) that is related to the overvaluation of body shape and weight [9, 10]. Although CBT is quite effective in BED, about 50% do not fully respond to treatment [11]. This may be related to the fact that overvaluation of body shape and weight is only present in a subset of individuals with BED [12]. In addition, dietary restraint seems to be stronger in bulimia nervosa than in BED [13, 14]. Interventions that focus on other maintaining mechanisms may therefore improve abstinence rates.

One model of interest is the affect regulation model. It assumes that binge eating is triggered by high levels of negative affect and that binge eating reduces negative affect [15, 16]. While mixed empirical support has emerged for the second part of this hypothesis (e.g. [17–20]), the first part has received extensive support from both retrospective studies (e.g. [17, 18, 21]), experimental studies [22] and ecological momentary assessment (EMA) studies [19, 20]. Also, greater elevations of negative affect prior to binge eating were found in BED when compared to bulimia nervosa [20]. Therefore, interventions that specifically target affect-related difficulties may improve outcome in patients with BED.

One treatment that specifically aims to address deficits in affect regulation is dialectical behavior therapy (DBT [23];). DBT, originally developed for patients with borderline personality disorder and ongoing self-harm or suicidal behaviors, has been adapted to treat BED (DBT-BED: e.g. [24]). DBT-BED aims to improve adequate emotion regulation skills in order to replace binge eating as a way of coping with negative affect [16]. To date, two randomized controlled trials have compared DBT-BED in patients with a primary diagnosis of BED to a waitlist control group, showing significantly less eating disordered behavior for DBT-BED at post-treatment and at 6 month follow-up [16, 25]. When compared to an active comparison group treatment (ACGT), post-treatment abstinence rates were favorable for DBT-BED (64% compared to 36% for ACGT), but there were no significant differences between the groups at any time during the 12-month follow-up period [26]. A fourth study [27] compared a more intensive version of DBT-BED to an adjusted, dose-matched, CBT-program in a mixed bulimia nervosa and BED sample of early weak responders to guided self-help cognitive behavior therapy. Although both treatments were helpful in reducing objective binge eating (OBE) episodes, no differences were found between treatments. These data support the idea that DBT can be a viable alternative to CBT in patients with binge eating. However, evidence is scarce and the available data do not favor one treatment over the other.

There are several reasons to assume that a certain subset of patients with BED is more likely to benefit from DBT. All eating disorders are characterized by emotion regulation difficulties, and although some studies suggest that individuals with BED may show these difficulties to a lesser extent than patients with anorexia nervosa or bulimia nervosa, patients with BED show marked emotion regulation difficulties when compared to healthy controls [28, 29]. Individuals who report to eat in response to negative emotions (emotional eating) have been shown to have higher levels of emotion regulation difficulties in comparison to groups without emotional eating [30]. Also, there is evidence suggesting that binge eating in overweight adults with BED is particularly associated with negative affect and not so much with dietary restraint (which is associated with binge eating in *normal weight* adults with BED [31–33]). Therefore, DBT might improve outcome in individuals with BED who are overweight and engage in emotional eating.

This study aimed to add to the current literature by comparing a DBT-BED group treatment to an intensive outpatient CBT-treatment (CBT+) in overweight individuals with BED who report above average levels of emotional eating. Although this CBT ‘treatment as usual’ comprised significantly more treatment time than the DBT intervention, and as such may have advantaged the CBT group, we hypothesized that DBT would be superior to CBT on measures related to eating disorder pathology and on measures related to emotion regulation. The reason for this is that we optimized chances for DBT-BED by including only individuals with BED who might be most likely to profit from an emotion regulation intervention.

## Method

### Study design

This study is an open, quasi-randomized, controlled trial with two arms: CBT+ and DBT-BED. When the study was designed, the CBT+ program had been treatment as usual at our center for 10 years. The program has shown to lead to substantial reductions in eating disorder pathology [34]. For pragmatic reasons we chose to compare DBT-BED with this more intensive program. We reasoned that, given the difference in dosage, we would be able to consider DBT-BED an important alternative to CBT+ if DBT-BED would at least equal the results of CBT+.

All patients that met the inclusion/exclusion criteria (described below) and provided written informed consent to participate in the study were allocated to either CBT+ or DBT-BED. An employee not involved in the clinical trial, randomized eligible patients by flipping a coin. If a treatment group was about to start with only one open slot, the patient to enter the study was assigned to that group rather than randomized. After allocation, participants completed assessments on the first day of treatment (baseline), on the last day of treatment (end of treatment) and 6 months after treatment (follow-up). Enrollment started in October 2011, and was finished by the end of 2016. The design of the study was approved on October 10th 2011 by the Institution of Mental Health Medical Ethics Committee (METiGG: 11.109; CMO Radboud UMC: 2013/226) and was registered retrospectively in the Netherlands Trial Register (NTR4154) on August 28, 2013. Prior to conducting any analyses, given the modest sample size, we made the decision to compare outcome between treatments only on core eating disorder variables.

### Participants

**Fig. 1:**
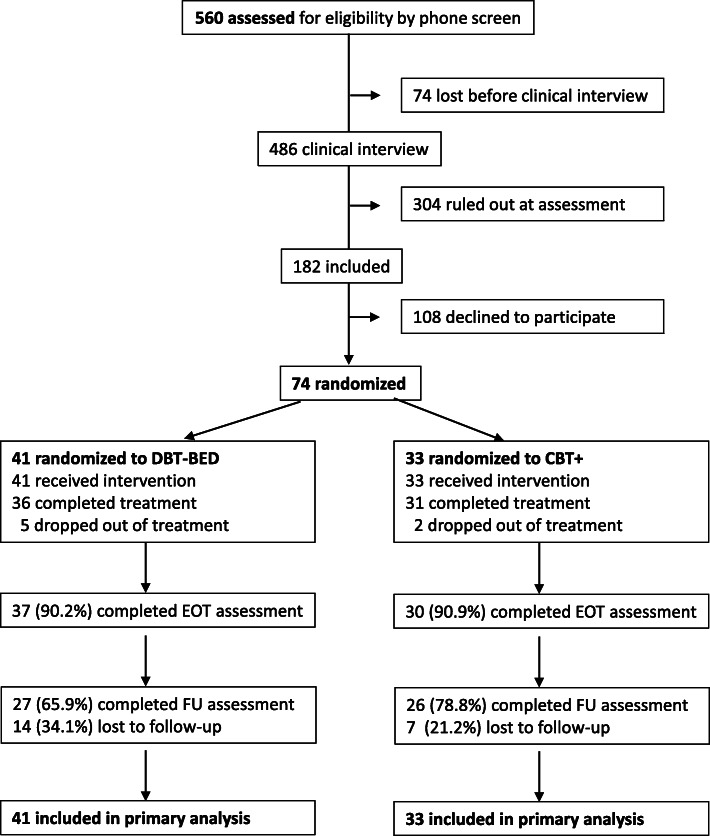
CONSORT flow diagram

Participants were individuals from 18 years upward, who were referred to an expertise center for eating disorders in the Netherlands. If, during a telephone screening, BED seemed plausible, patients were asked to fill out the Dutch Eating Behavior Questionnaire (DEBQ [35];). Subsequently, either a licensed psychologist or psychiatrist conducted a clinical interview, designed a case formulation and determined the presence or absence of BED according to DSM-5 [1]. The case formulation and the DSM-5 classification were then reviewed in a multidisciplinary team. Because individuals with BED who are overweight and engage in emotional eating are arguably most likely to benefit from an emotion regulation intervention, we only included patients with a BMI ≥ 30 and an above average urge to eat in response to negative emotions (score ≥ 2.38 on the DEBQ subscale Emotional Eating [36];). Exclusion criteria were kept to a minimum to ensure generalization of study results: previous CBT treatment for being overweight or eating disorder; current substance abuse, psychosis, suicidality; severe personality disorder; obesity caused by physical illness; concurrent treatment for being overweight or for eating disorder by medical specialist or dietician. Eligible patients were informed about the study by the clinician who conducted the initial clinical interview. They were given written information together with an informed consent form. All questions were answered. They then were asked to send back a signed form within 2 weeks. During this period a member of the research team was available for additional questions (Fig. 1).

### Treatment

#### Dialectical behavior therapy for binge eating disorder (DBT-BED)

A Dutch prepublication version of the DBT-BED session-to-session protocol (courtesy of C. Telch and D. Safer [24]) was used. DBT-BED teaches skills to help patients regulate emotions in an adaptive way. This is done from a ‘dialectical’ stance: accept patients as they are and at the same time stimulate them to change in order to help them reach their goals. Treatment included 20 group-sessions of 2 h each, over the course of 20 weeks. In the first two sessions the rational and the goals of therapy were reviewed comprehensively, and an explicit commitment to change was made. The use of diary cards and chain analyses was introduced as well as the concept of therapy interfering behavior. The second phase (sessions 3–18) comprised three modules in which skills in mindfulness, emotion regulation and distress tolerance were taught. The emotion regulation module incorporates lifestyle interventions (i.e. education on a balanced eating pattern and regular physical exercise) to diminish the sensitivity for negative emotions. The third phase focused on the review and enhancement of learned skills, and on plans for the future. During treatment, patients monitored their weight weekly at the treatment center in order to help them face the consequences of (changes in) their eating behavior. A maximum of nine patients could participate in each round, in a closed group format. Six months after the end of treatment, progress was reviewed and skills were refreshed in a single follow-up group session. At that time, further treatment was offered in case this seemed necessary. Each treatment-cycle of 20 weeks was led by two trained psychologists/psychotherapists. Several therapist-pairs were formed as the treatment was provided over a substantial period of time.

#### Intensive outpatient cognitive behavior therapy (CBT+)

The CBT intervention was an extended version in group format of the manual developed by Fairburn and colleagues [37], addressing binge eating as behavior maintained by dietary restraint and other behavior, like body avoidance, originating from the overvaluation of weight and shape. The session-to-session protocol is available from ML. Treatment included 20 days of group therapy, 1 day per week during 20 consecutive weeks. Each day comprised three modules of 75 min each: 1) discuss daily self-monitoring of eating behavior and related situations, thoughts and feelings, 2) challenge thoughts and conduct behavioral experiments related to food and eating, and 3) challenge thoughts and conduct behavioral experiments related to body image. Over the course of treatment, several topics were covered in all three modules: motivation to change, eating regularly and sufficiently, dealing with triggers for binge eating (including some emotion regulation strategies), body image, body satisfaction, life-style and relapse prevention. In addition, patients monitored their weight weekly at the treatment center in order to diminish the obsession with weight or to break the avoidance of weight, and to monitor the consequences of (changes in) eating behavior. Each treatment-cycle of 20 weeks was led by a team of three: a psychologist, a psychiatric nurse and a psychomotor therapist. Psychomotor therapists addressed maintaining factors like body-avoidance and comparison-making by in-session exercises (e.g. body-exposure) and related homework assignments (see also [38]). Several therapist-combinations were formed as the treatment was provided over a substantial period of time. A maximum of nine patients could participate in each round. New patients entered every 10th week (i.e. a half open group format). In addition, six group meetings of 90 min each were offered to patients and their partners to enhance mutual understanding and support during the process of change. After treatment, if deemed necessary on clinical grounds, six monthly group sessions were offered to help prevent relapse.

#### Therapist qualifications/training

All therapists were well trained and experienced in CBT for eating disorders, as this is the treatment as usual at the treatment center. Training in DBT-BED was initially provided by a senior psychologist, independent from this study, and well trained in this specific protocol. Later on, the initially trained therapists trained the co-therapist. Therapists in the DBT-BED-condition were supervised once a month by a leading expert in DBT. Therapists in the CBT-condition did not receive supervision, although peer consultation was ensured. No therapist worked in two treatments at the same time in order to avoid content or procedural overlap. Treatment adherence was assessed (see Supplementary material).

#### Assessment

Except for demographic information and height (collected at baseline only), all assessment instruments were administered at baseline, at post-treatment and at follow-up. All the assessed psychopathology measures were self-report questionnaires. Staff conducting the assessments was aware of the treatment condition that patients were assigned to.

##### Eating disorder pathology

The Eating Disorder Examination Questionnaire (EDE-Q [39]) was used to assess the number of OBE episodes and global levels of eating disorder psychopathology over the past 28 days. Higher scores indicate greater severity. The EDE-Q is considered reliable for patients with BED [40] and has acceptable to high internal consistency and overall test-retest reliability [41]. However, empirical support for the subscales (restraint, eating concern, shape concern and weight concern) is questionable [42, 43].

##### Emotion regulation

The urge to eat in response to negative emotions was assessed with the 13-item subscale Emotional Eating of the Dutch Eating Behavior Questionnaire (DEBQ [35, 36]). Higher scores indicate higher levels of emotional eating. The reliability and validity of the DEBQ are rated as good (enough) and all subscales have good internal consistency and factorial validity (e.g. [44, 45]).

The subscale Emotional Dysregulation of the Eating Disorder Inventory (EDI-3 [46]) was used to assess the tendency toward poor impulse regulation and mood intolerance. The EDI-3 assesses psychological and behavioral eating disorder symptomatology. Higher scores indicate more psychopathology. The reliability and the validity of the EDI-3 are considered to be good for use in eating disorder patient groups [47].

##### General psychopathology

General psychopathology was measured using the total score of the Symptom Checklist 90 (SCL-90). The SCL-90 consists of 90 items related to the frequency of experienced physical and psychological complaints in the last week. Higher sum scores reflect more general psychopathology. The reliability and validity of the SCL-90 are good [48].

The Beck Depression Inventory-II (BDI-II) consists of 21 questions about the severity of depressive symptoms in the last week. Higher sum scores indicate more depressive symptoms. The reliability and validity of the BDI-II are good [49]. Self-esteem was assessed with the EDI-3 subscale Low Self-Esteem [46].

##### Weight, body mass index

Patients were measured for height and weight, through which we computed their BMI: kg/m^2^. Patients were measured for weight on a balanced scale wearing cloths but no shoes.

##### Dropout

Dropout was defined as premature termination of treatment, either patient-initiated or staff-initiated. Patients were allowed to miss a maximum of 2 out of 20 days. If they missed more, they were excluded from treatment and were consequently considered dropout of treatment. When treatment was terminated before the 20-week period ended because staff and patient mutually agreed that treatment goals were achieved, this was considered as completion of treatment instead of dropout.

### Power and sample calculation

Initial power analysis suggested that a sample of 34 per group (68 total) would provide a power of .80 to detect a medium effect. A total of 74 participants (33 to CBT+ and 41 to DBT-BED) were randomized. At the conclusion of the trial, it was discovered that the initial power analysis was incorrect. The actual power to detect a medium effect based upon an alpha of .05 is only .58.

### Statistical analysis

All analyses were conducted using SPSS Version 25 [50]. Significance tests were based on a two-tailed alpha of 0.05. Primary measures of outcome used to evaluate efficacy included OBE episodes and EDE-Q Global scores. Secondary measures of outcome included DEBQ Emotional Eating, EDI-3 Emotional Dysregulation, SCL-90 total score, BDI-II total score, and EDI-3 Low Self-Esteem. Distribution diagnostics for primary and secondary outcome measures suggested that all outcome measures except OBE episodes were symmetrically distributed and appropriate for normal assumption models. CBT+ and DBT-BED treatment groups were compared separately at end of treatment and follow-up controlling for baseline assessment using a generalized linear model with a negative binomial distribution for OBE episodes and a general linear model for all other outcome variables. Given that treatment for both CBT and DBT was delivered in group settings, preliminary models were run nesting participants within therapeutic groups. As no significant variation attributable to therapeutic group was found, subsequent analyses were conducted without nesting. Final models included a main effect for treatment group, and a fixed covariate for baseline assessment. Effect sizes between treatments were calculated using both Cohen’s [51] *d* and the *success rate difference* (*SRD* [52]). Cohen’s *d* values were calculated from covariate-adjusted estimated marginal means; Cohen uses values of 0.2, 0.5, and 0.8 to characterize “small”, “medium”, and “large” differences between groups, respectively. *SRD* values, which can range from − 1 to + 1, represent the probability that a randomly selected case from one treatment will have an outcome preferable to a randomly selected case from another treatment.

Outcome analyses were based upon the intention-to-treat principle [53]. Multiple imputation was used to impute missing data using fully conditional Markov chain Monte Carlo (MCMC [54]) modeling. The final analyses were based upon the pooled results of 20 separate imputation sets. Sensitivity analyses were conducted using both maximum likelihood imputation and available data analyses to evaluate the consistency of results across differing methods for handling missing data.

Clinically meaningful change was operationalized as proposed by Jacobson & Truax [55]. We calculated the percentage of patients on the EDE-Q Global score that shifted from being closer to the mean of the dysfunctional group (current sample: mean = 3.29; SD = .959) to being closer to the mean of a functional group (i.e. a normative non-students sample of males and females from the United Kingdom: mean = 1.92; SD = 1.42 [56]).

## Results

### Study participants

A total of 74 participants were randomized: 33 to CBT+ and 41 to DBT-BED. Participants included 66 (89.2%) women and 8 (10.8%) men, with an average age of 37.3 (SD = 11.8; range = 18–67) and an average duration of illness of 15.3 years (SD = 10.9; range = 1–45). BMI of participants averaged 39.9 (SD = 5.6; range = 30.5–55.5). The majority of participants (*n* = 49 [66.2%]) lived with a partner/spouse. Treatment groups did not differ significantly on any demographic characteristics, BMI, or outcome measures at baseline.

### Study retention

A total of 7 (9.5%) participants dropped out of the treatment and/or study during the course of the trial, including 2 (6.1%) from CBT+ and 5 (12.2%) from DBT-BED (Fisher’s Exact *p* = .451). Of the 74 participants that were randomized, 67 (90.5%) completed end of treatment assessments and 53 (71.6%) completed follow-up assessments. Assessment completion rates for CBT+ and DBT-BED were 90.9% vs. 90.2% (Fisher’s Exact *p* = 1.00) respectively at end of treatment and 78.8% vs. 65.9% (Fisher’s Exact *p* = .301) at follow-up.

### Primary outcomes

Mean scores on primary measures of outcome for CBT+ and DBT-BED groups at baseline, end of treatment, and follow-up are presented in Table 1. The CBT+ group experienced greater reductions in EDE-Q Global score that approached significance at end of treatment (*p* = .060) and reached significance at follow-up (*p* = .020), with effect sizes ranging from 0.45 at end of treatment to 0.55 at follow-up. Results of sensitivity analyses using maximum likelihood (ML) imputation and available data (AD) analysis produced relatively consistent results at end of treatment (ML: *p* = .050; AD: *p* = .052) and follow-up (ML: *p* = .006; AD: *p* = .006). Table 2 presents the percentage of participants who completed the EDE-Q that shifted from a dysfunctional level at baseline to a functional level at end-of-treatment and follow-up (cut-off EDE-Q score: 2.47 [55]). Although percentages were higher for CBT+ at both end-of-treatment and follow-up (69.6 and 65.0% vs 52.9 and 45.8% for DBT-BED), these differences were not significant.

**Table 1:** CBT+ vs. DBT-BED Comparison of Treatment Outcome

Outcome	Group		Study Visit (mean, SD)			CBT+ vs. DBT-BED					
		N				EOT			FU		
			Baseline	EOT	FU	Sig.	*d*	*SRD*	Sig.	*d*	*SRD*
EDE-Q Global^a^	CBT+	33	3.06 (1.10)	1.64 (1.16)	1.61 (1.11)	.060	.45	.248	.020	.55	.302
	DBT-BED	41	3.48 (0.79)	2.31 (1.09)	2.35 (1.06)						
OBE Episodes^a^	CBT+	33	8.27 (9.65)	0.74 (1.68)	1.85 (5.11)	.035	.46	.253	.095	.37	.204
	DBT-BED	41	7.51 (8.72)	1.64 (3.77)	2.75 (5.58)						
DEBQ Emotional Eating^b^	CBT+	33	3.76 (0.69)	2.55 (0.64)	2.45 (0.86)	.322	.23	.128	.196	.29	.161
	DBT-BED	41	3.77 (0.68)	2.72 (0.64)	2.73 (0.83)						
EDI-3 Emotional Dysregulation^b^	CBT+	33	25.09 (6.80)	21.84 (3.72)	21.23 (4.61)	.392	.21	.117	.253	.27	.150
	DBT-BED	41	27.02 (5.93)	23.81 (6.74)	22.88 (3.76)						
SCL-90^b^	CBT+	33	175.5 (51.9)	136.0 (39.6)	128.8 (37.1)	.257	.27	.150	.152	.34	.188
	DBT-BED	41	185.9 (43.1)	150.7 (45.4)	144.3 (38.4)						
BDI-II^b^	CBT+	33	20.53 (9.89)	7.56 (6.52)	7.21 (6.45)	.193	.31	.172	.098	.39	.215
	DBT-BED	41	21.98 (7.60)	10.69 (8.46)	10.75 (8.20)						
EDI-3 Self-Esteem^b^	CBT+	33	35.44 (9.00)	25.89 (7.96)	24.12 (8.17)	.072	.43	.237	.014	.59	.324
	DBT-BED	41	38.32 (8.47)	30.80 (9.64)	29.75 (8.00)						

*EOT* End of treatment, *FU* Follow-up, *d* Cohen’s *d*, *SRD* Success rate difference

^a^Primary outcome measure

^b^Secondary outcome measure

**Table 2:** Percentage of Participants that went from above to below the Cutoff of 2.47 on the EDE-Q Global Score

	CBT+	DBT-BED	Fisher’s Exact p
**End of Treatment**	69.6% (16/23)	52.9% (18/34)	.275
**Follow-up**	65.0% (13/20)	45.8% (11/24)	.238

The CBT+ group also showed greater reductions in OBE episodes at end of treatment (*p* = .035; *d* = .46), but these differences were no longer significant at follow-up (*p* = .095). The end of treatment differences between treatment groups in OBE episodes were confirmed in sensitivity analyses (ML: *p* = .010; AD: *p* = .010).

### Secondary outcomes

Results of secondary outcome analyses are presented in Table 1. *SRD*s show preferable probability of improvement for CBT+ on all secondary measures at both end of treatment and follow-up; however, the only difference in secondary outcome measures that reached significance was for EDI-3 Low Self-Esteem. The CBT+ group experienced greater reductions in EDI-3 Low Self-Esteem that approached significance at end of treatment (*p* = .072; *d* = .43) and reached significance at follow-up (*p* = .014; *d* = .59). Results of sensitivity analyses confirmed these findings at both end of treatment (ML: *p* = .053; AD: *p* = .064) and follow-up (ML: *p* = .024; AD: *p* = .018).

### Treatment adherence

Mean session integrity was 79.1% (SD = 15.0) for DBT-BED and 63.5% (SD = 24.1) for CBT+ with a statistically significant difference in favor of DBT (*p* < .001). To establish interrater reliability, five raters rated four tapes independently. The average kappa coefficient across raters and tapes was .628 (*p* < .001) suggesting good agreement.

## Discussion

This controlled study compared an emotion regulation treatment adjusted for BED with an intensive eating disorders-focused form of CBT in obese individuals with BED. Contrary to our expectations, DBT-BED was not superior to and was in fact less efficacious than CBT+ on primary outcome measures, especially on reductions in eating disorder psychopathology at follow-up. The greater reductions in OBE episodes in CBT+ at end of treatment were not retained 6 months after treatment. Reductions on all secondary measures were consistently in favor of the CBT+ group, with self-esteem reaching statistical significance and a medium effect at follow-up.

The failure to support our primary hypothesis may be due to differences in dosage between treatments: CBT+ contained more face-to-face contact time per day (3.75 h versus 2 h per week), offered six group meetings of 90 min to patients with a partner and incorporated six follow-up sessions for some patients (versus one for all patients in DBT-BED). Thus, this latter group received twice-weekly sessions during 6 weeks. Dose-response research in psychotherapy shows that, more than the number of sessions and the total contact time, the frequency of treatment schedules seems to be a relevant factor as more frequent treatment schedules (e.g. twice per week instead of once per week) are found to be more effective [57–59]. Therefore, patients in CBT+, especially those that participated in the ‘partner-group’, have this advantage.^1^ As Chen and colleagues [27] found no superiority of either a more intensive DBT-program or a dose-matched CBT-program, the DBT-BED program in this study may possibly have been as efficacious as CBT if CBT was dose-matched.

Also, again contrary to our expectations, we did not detect any differences between DBT-BED and CBT+ at end of treatment or follow-up on measures related to emotion regulation. This seems remarkable given the theoretical foundation of both therapies with DBT-BED targeting emotion regulation and CBT targeting dietary restraint and other behavior originating from the overvaluation of weight and shape. Possible reasons for failing to find differences may be related to limited statistical power or to increased treatment time in CBT+. Concurrently, to stay close to clinical practice we did not control for content and therefore conceptual overlap may have occurred. Differential effects of both therapies were possibly compromised because of this. However, it should be noted that findings on the emotion regulation measures in this study are in line with Safer and colleagues [26] who found a consistent lack of differential impact with a broad range of emotion-regulation measures comparing DBT-BED to an active controlled for content comparison. Also, in individuals with bulimia nervosa, CBT has been found to produce decreases in emotion dysregulation [60]. This suggests that decreases in emotion dysregulation might not be attributable to the specific emotion regulation techniques used in DBT-BED, but to therapeutic elements shared across various treatments.

Apart from self-esteem, no significant differences in reduction between the groups were found on measures related to general psychopathology. Depressive features improved considerably in both groups.

Improvements in OBE episodes seemed to diminish slightly between end of treatment and follow-up in both groups but stayed, on average, below the diagnostic threshold (< 4 OBE episodes in 28 days). This is in line with previous findings (e.g. [27]). In addition, a substantial percentage of patients in both groups reached clinically meaningful change in eating disorder psychopathology. Percentages were higher for CBT+ at both end-of-treatment and follow-up, but these differences were not significant.

The study has several limitations. One major limitation of this study is the difference in dosage between DBT and CBT+, which compromises our ability to draw solid conclusions about the observed differences in outcome. A second major limitation is the limited sample size. With higher power we may have found more, and more robust, differences in favor of CBT+, as all non-significant differences favored CBT+. However, despite the power issues, we did find significant differences between treatments on both primary measures. Also, treatment adherence was lower in CBT+, possibly due to the fact that therapists received no supervision. Since higher adherence levels are related to better outcome [61, 62], differences in outcome between the two treatments may even have been bigger had the adherence to the CBT+ protocol been higher.

We could have further optimized the assessment of BED pathology by either making use of the Eating Disorder Examination interview (EDE) instead of the EDE-Q [41] or by providing a specific definition of binge eating when administering the EDE-Q (as suggested by Celio and colleagues [63]). In addition, we did not control for content which may have compromised differential effects of both therapies. Finally, allocation was strictly not entirely random.

Despite the limitations, the present study has several strengths. To our knowledge, this is the first controlled study in individuals with BED, comparing DBT-BED, previously tested only against a waitlist [16, 25] and an active comparison [26], to a CBT-program. This study is therefore a step forward in evaluating the efficacy of DBT-BED. The selection of a subgroup of BED patients (obese BED patients who report an above average urge to eat in response to negative emotions) optimized the chances of DBT-BED to prove itself as a viable alternative to an intensive outpatient CBT program. Further, although dropout rates in DBT-BED (17.1%) were relatively high when compared to Safer and colleagues [26] (4%), dropout rates in CBT+ (6%) were low when compared to other controlled CBT-treatment studies (e.g. 16.7 to 30% [64, 65]). Besides that, generalizability was optimized by conducting the study in routine clinical practice, with few exclusion criteria, and making use of various therapist-pairs (which enables us to generalize beyond the present therapist sample). Finally, the follow-up period provides insight in the medium-long term effects of both treatments.

**In conclusion**, the more intensive CBT+ reduced eating disorder related measures and self-esteem more than DBT-BED, even in a population that arguably may be more likely to profit from an emotion regulation intervention. This clearly favors CBT+ above DBT-BED. Yet, when looking at outcome from a different perspective, the data suggest that both groups reached comparable levels of clinically meaningful change in global eating disorder psychopathology. This is particularly interesting given that the DBT-BED program is less time-consuming so less costly than CBT+ as applied in the current study. To be able to fully understand the value of DBT-BED, future research should include dose-matched comparisons of CBT and DBT-BED in a sufficiently powered randomized controlled trial and include longer term follow-up. Furthermore, maybe even more important, future studies should search for mediators and moderators to improve outcome in current efficacious treatments for BED.

## Supplementary information

**Additional file 1.**

## Data Availability

The datasets used and/or analyzed during the current study are available from the corresponding author on reasonable request.

## Abbreviations

ACGT: Active Comparison Group Therapy
AD: Available Data
BED: Binge Eating Disorder
BDI-II: Beck Depression Inventory-II
BMI: Body Mass Index
CBT: Cognitive Behavioral Therapy
CBT +: Intensive outpatient group CBT-treatment
CMO: Commissie Mensgebonden Onderzoek
COTAN: Commissie Testaangelegenheden Nederland [Dutch committee on tests and testing]
DBT: Dialectical Behavior Therapy
DBT-BED: Dialectical Behavior Therapy adapted for Binge Eating Disorder
DSM-5: Fifth edition of the Diagnostic and Statistical Manual of Mental Disorders
DEBQ: Dutch Eating Behavior Questionnaire
EDE-Q: Eating Disorder Examination Questionnaire
EDI-3: Eating Disorder Inventory-3
MCMC: Markov Chain Monte Carlo
METiGG: Institution of Mental Health Medical Ethics Committee
ML: Maximum Likelihood
NTR: Netherlands Trial Register
SCL-90: Symptom Checklist-90
SD: Standard Deviation
SRD: Success rate difference
UMC: University Medical Center

## Footnotes

1 On the other hand, one could also say that we have advantaged DBT-BED as we selected a sample that we assumed could optimally benefit from the DBT-BED treatment.
