# Correction to: Dialectical behavior therapy adapted for binge eating compared to cognitive behavior therapy in obese adults with binge eating disorder: a controlled study

**DOI:** 10.1186/s40337-021-00515-4

**Published:** 2021-12-21

**Authors:** Mirjam W. Lammers, Maartje S. Vroling, Ross D. Crosby, Tatjana van Strien

**Affiliations:** 1 Amarum, Expertise Centre for Eating Disorders, GGNet Network for Mental Health Care, Den Elterweg 75, 7207 AE Zutphen, The Netherlands; 2 Radboud University, Behavioural Science Institute, Montessorilaan 3, 6525 HR Nijmegen, The Netherlands; 3 Sanford Center for Biobehavioral Research, Fargo, North Dakota USA; 4 University of North Dakota School of Medicine and Health Sciences, Fargo, North Dakota USA

## Correction to: Journal of Eating Disorders (2020) 8:27 10.1186/s40337-020-00299-z

Unfortunately, the original version of this article [1] contained errors. After publication it came to the authors’ attention that we used the wrong scoring for two the of secondary outcome measures: EDI-3 Emotional Dysregulation and EDI-3 Self-Esteem. In addition, by mistake, CBT+ and DBT were switched in the Abstract description of the results regarding clinically significant change.

In the analyses that were published, there were significant differences on Low Self-Esteem at follow-up favoring CBT+, but no other differences in both Emotional Dysregulation and Low Self-Esteem. With the correct scoring we found significant differences favoring CBT+ on Emotional Dysregulation at end of treatment and Self-Esteem at both end of treatment and follow-up. Our conclusion favoring CBT+ has not changed, but the evidence supporting this conclusion has strengthened.

The **Abstract** should report that:

**Results:** Overall, greater improvements were observed in CBT+. Differences in number of objective binge eating episodes, emotional dysregulation (EDI-3 Emotional Dysregulation) and self-esteem (EDI-3 Low Self Esteem) at end of treatment, and eating disorder psychopathology (EDE-Q Global score) and self-esteem at follow-up reached statistical significance with small to medium effect sizes (Cohen’s *d* between 0.43 and 0.66). Of the patients in the CBT+ group, 69.9% reached clinically significant change at end of the treatment vs 65.0% at follow-up. Although higher, this was not significantly different from the patients in the DBT group (52.9% vs 45.8%).

**Plain English Summary:** Greater improvements were observed in the CBT group regarding the number of objective binge eating episodes, emotional dysregulation and self-esteem at the end of treatment, and regarding global eating disorder psychopathology and self-esteem 6 months after treatment.

Table 1 should report:

**Table 1:** CBT+ vs. DBT-BED comparison of treatment outcome

Outcome	Group	N	Study visit (mean, SD)			CBT+ vs. DBT-BED					
						EOT			FU		
			Baseline	EOT	FU	Sig.	*d*	*SRD*	Sig.	*d*	*SRD*
EDE-Q global^1^	CBT+	33	3.06 (1.10)	1.64 (1.16)	1.61 (1.11)	.060	.45	.248	.020	.55	.302
	DBT-BED	41	3.48 (0.79)	2.31 (1.09)	2.35 (1.06)						
OBE episodes^1^	CBT+	33	8.27 (9.65)	0.74 (1.68)	1.85 (5.11)	.035	.46	.253	.095	.37	.204
	DBT-BED	41	7.51 (8.72)	1.64 (3.77)	2.75 (5.58)						
DEBQ emotional eating^2^	CBT+	33	3.76 (0.69)	2.55 (0.64)	2.45 (0.86)	.322	.23	.128	.196	.29	.161
	DBT-BED	41	3.77 (0.68)	2.72 (0.64)	2.73 (0.83)						
EDI-3 emotional dysregulation^2^	CBT+	33	5.09 (4.50)	2.55 (2.19)	2.38 (2.20)	.038	.43	.239	.437	.29	.162
	DBT-BED	41	5.59 (3.64)	3.94 (3.91)	2.99 (1.98)						
SCL-90^2^	CBT+	33	175.5 (51.9)	136.0 (39.6)	128.8 (37.1)	.257	.27	.150	.152	.34	.188
	DBT-BED	41	185.9 (43.1)	150.7 (45.4)	144.3 (38.4)						
BDI-II^2^	CBT+	33	20.53 (9.89)	7.56 (6.52)	7.21 (6.45)	.193	.31	.172	.098	.39	.215
	DBT-BED	41	21.98 (7.60)	10.69 (8.46)	10.75 (8.20)						
EDI-3 self-esteem^2^	CBT+	33	11.13 (5.55)	5.55 (4.39)	4.93 (4.59)	.047	.56	.308	.033	.66	.359
	DBT-BED	41	12.83 (4.98)	8.48 (5.89)	8.03 (4.84)						

*d* = Cohen’s *d*; *SRD* = success rate difference; *EOT* = end of treatment; *FU* = follow-up

^1^Primary outcome measure

^2^Secondary outcome measure

The **Secondary Outcomes** should report:

Results of secondary outcome analyses are presented in Table 1. SRDs show preferable probability of improvement for CBT+ on all secondary measures at both end of treatment and follow-up; differences in secondary outcome measures that reached significance were for EDI-3 Emotional Dysregulation and for EDI-3 Low Self-Esteem. The CBT+ group experienced greater reductions in EDI-3 Emotional Dysregulation at end of treatment (*p* = 0.038; *d* = 0.43). Also, the CBT+ group experienced greater reductions in EDI-3 Low Self-Esteem at both end of treatment (*p* = 0.0.47; *d* = 0.56) and at follow-up (*p* = 0.033; *d* = 0.66). Results of sensitivity analyses confirmed these findings at both end of treatment (ML: *p* = 0.048; AD: *p* = 0.051) and follow-up (ML: p = 0.037; AD: *p* = 0.022).

The **Discussion** should report:

Also, again contrary to our expectations, we did not detect any differences in favor of DBT-BED on measures related to emotion regulation. Indeed, at end of treatment, CBT+ outperformed DBT-BED on emotional dysregulation. This seems remarkable given the theoretical foundation of both therapies with DBT-BED targeting emotion regulation and CBT targeting dietary restraint and other behavior originating from the overvaluation of weight and shape. Possible reasons for failing to find differences may be related to limited statistical power or to increased treatment time in CBT+. Concurrently, to stay close to clinical practice we did not control for content and therefore conceptual overlap may have occurred. Differential effects of both therapies were possibly compromised because of this. However, it should be noted that most findings on the emotion regulation measures in this study are in line with Safer and colleagues [26] who found a consistent lack of differential impact with a broad range of emotion-regulation measures comparing DBT-BED to an active controlled for content comparison. Also, in individuals with bulimia nervosa, CBT has been found to produce decreases in emotion dysregulation [60]. This suggests that decreases in emotion dysregulation might not be attributable to the specific emotion regulation techniques used in DBT-BED, but to therapeutic elements shared across various treatments.
