# Supplementary material for: Dialectical behavior therapy adapted for binge eating compared to cognitive behavior therapy in obese adults with binge eating disorder: a controlled study
Source: J Eat Disord. 2020 Jun 10;8:27. doi: 10.1186/s40337-020-00299-z (PMC7285554; doi:10.1186/s40337-020-00299-z)
Supplement: Supplementary file 1 — Additional file 1. [file 40337_2020_299_MOESM1_ESM.docx]

Supplementary material

**Treatment adherence**

All treatment sessions were audio-recorded. A random sample of 10% of all DBT sessions and 10% of all CBT module 2 sessions were rated for treatment adherence by five independent raters (Masters-level doctoral students in psychology). We constructed a format based on the DBT and CBT manuals. Each treatment session was broken down into the specific components of that session. The raters assessed whether or not the actually discussed components corresponded to the format. The percentage agreement between the format and the specific session was used to establish session integrity. Besides that, two DBT-BED and two CBT-sessions were rated by all raters involved in order to assess interrater reliability. Raters were trained by ML.
